# Precision Oncology in Resource-Constrained Settings: Challenges and Ethical Dilemmas in Low- and Middle-Income Countries

**DOI:** 10.7759/cureus.115830

**Published:** 2026-09-05

**Authors:** Zubair Ahmed, Arham Amir Khawaja, Aiman Tariq, Haseeb Mehmood Qadri

**Affiliations:** 1 Pediatrics and Neonatology, Shaikh Zayed Medical Complex, Lahore, PAK; 2 General Surgery and Surgical Oncology, Shaikh Zayed Medical Complex, Lahore, PAK; 3 Internal Medicine, Sir Ganga Ram Hospital, Lahore, PAK; 4 General Surgery, Lahore General Hospital, Lahore, PAK; 5 Neurological Surgery, Punjab Institute of Neurosciences, Lahore, PAK

**Keywords:** genetic therapy, genome, human, medical oncology, mutation, neoplasms, precision medicine, technology

## Abstract

Human genomes have been explored in an elaborate fashion. However, the road to understanding genomics-guided treatment is still in its infancy. Genome-wide sequencing studies have identified genetic variants and somatic mutations that lead to cancer. Precision oncology uses genomic and molecular information to guide individualised therapeutic strategies tailored to each cancer patient. This approach requires distinguishing somatic tumour profiling from germline testing, as their implications for consent, disclosure, confidentiality and the identification of inherited cancer susceptibility differ. The stark contrast in the application of precision cancer medicine (PCM) in low- and middle-income countries (LMICs) versus high-income countries (HICs) has led to difficulty in adopting this concept and, in the long run, the application. PCM is of paramount importance for achieving therapeutic evidence for cancer by analysing and creating molecular targets for specific genes. Although this emerging field is progressively becoming a footprint of medical oncology, many challenges still exist that could hinder the success of precision oncology in LMICs. The disparity in the introduction of precision oncology in LMICs needs a different approach, considering the structural, ethical, socioeconomic and cultural barriers.

## Editorial

Advances in genetic studies have transformed cancer care by enabling comprehensive characterisation of the genomic landscape, facilitating more personalised approaches to cancer diagnosis, prognosis and treatment. It has enabled clinicians, researchers and analysts to implement targeted therapies for cancer management. The increasing burden of cancer-related mortality has led to a paradigm shift in cancer care, with advances in molecular genetics enabling more targeted approaches [1]. A holistic search of genomic profiling has expanded the evidence base, facilitating the integration of targeted therapies into clinical practice. The generated data are still deemed to warrant exploration owing to unsolicited findings that require clinical interpretation regarding heredity and treatment futility. Furthermore, it has spotlighted a few ethical dimensions with the discovery of these unknown secrets and the expansion of genomic data use worldwide. This targeted precision oncology is a long road, and it is still just the tip of the iceberg. The ongoing challenges and continual evolution of human responses required to address them will unfold in due course according to the population characteristics. It is important to anticipate the responses, especially given that precision oncology is still an emerging field.

Genetics has progressed over a decade from linked marker analysis to whole-genome testing with the notion of providing personalised care for a variety of diverse diseases, including both rare and common disorders. The worldwide use of genomic technology poses several financial, sociocultural, religious and legislative constraints, including lack of readiness to accept and integrate this innovation into the current clinical framework. There are global inequalities and challenges borne by low- and middle-income countries (LMICs) as their health systems face profound challenges in implementing comprehensive cancer control programmes because of limited health care resources and infrastructure and genomic underrepresentation [2]. These barriers hinder the adoption of precision medicine among those most in need. Therefore, it has become a privileged tool rather than one for public health use. These, in addition to limited knowledge among healthcare practitioners and the public and hesitancy towards technology adoption, have contributed to suboptimal uptake and persistent underrepresentation in genomic datasets. Additionally, part of this disparity comes from the large volume of research, approximately 86.3% of genome-wide association studies being carried out in people of European ancestry [3]. This underrepresentation has significant consequences in oncology, where genetic interpretations are often less accurate in non-European populations. Therefore, guidelines generated in high-income countries (HICs) may not be implemented in LMICs.

The integration of this novel intervention requires a structured framework, a trained and well-educated genomic workforce, including counsellors and geneticists, established algorithms, follow-up strategies and data-handling policies and laws to maintain security. Echinger et al., in their scoping review, echoed the wide variety of ethical issues which may arise due to the lack of the above essential components [4]. The potential lack of well-trained genomic professionals in LMICs may hinder the provision of informed and valid consent. Hence, defining the core concepts necessary for informed consent for genetic testing provides a foundation for quality patient care across a variety of healthcare providers and clinical indications [5]. A clear distinction between somatic and germline mutations is essential for informed consent. Somatic testing identifies tumour-specific mutations to guide treatment, while germline testing detects inherited variants and requires separate consent due to implications for family members and unexpected findings. These unsolicited findings may unfold due to sequencing artefacts, unknown genetic variants, inadequate tissue sampling and false-positive results of biopsy. These incidental or secondary outcomes of germline testing may emerge as therapeutic misconceptions, public mistrust, and affect individuals’ social identity by labelling them as diseased or disabled, which will require confirmatory testing and genetic counselling before guiding clinical or family decisions. This may increase psychological frustration, unemployment, discrimination and unequal benefits in society, thus increasing poverty and possibly the crime rate. The usage of sensitive personal and community data can cause harm and insecurity, as it will generate stigma and skew care prioritisation.

There can be an issue of potentially widening health disparities, as LMICs mostly struggle with basic care; acceptance of this expensive technology will become a problem. The use of this testing will strain a country's economy and make it more dependent on international funding and resources. Therefore, a reasonable approach will be to scale the PCM implementation to match the existing infrastructure of LMICs. It should be employed in a staged manner: initially strengthen the essential pathology and testing for high-yield mutations only, followed by focused panels for selected cancers where cost-effective targeted therapies, quality data handling and follow-up are essentially available. Additionally, create regional molecular tumour boards that bring together genomics experts from different hospitals or countries to help interpret test results and guide treatment decisions. This will help prevent data generation, which cannot be acted upon. Conventional health technology assessment approaches may be insufficient because they do not adequately account for differences in the capacity to pay. From an economic perspective, the global implementation of precision oncology must address disparities in accessibility, affordability and health-system readiness. Low- and middle-income countries frequently face limited or delayed access to novel targeted therapies, further widening the global inequities in precision cancer care [6].

The guidelines of most developed countries ethically obligate physicians to follow the patient-centred approach, keeping in view the due autonomy of an individual. In many LMICs, counselling practices may deviate from this standard, with some clinicians adopting a more directive style and, in certain instances, prioritising family or caregiver preferences over the patient’s interests; a pattern that, in its most extreme form, echoes historically eugenic reasoning and warrants caution. Hence, there may be an inclination among caregivers to rely on clinicians to make decisions, with little desire to learn more about treatment options [7]. In addition to over-reliance, a misconception that testing guarantees definitive treatment underscores the need for sustained educational investment to strengthen multidisciplinary teamwork and ethical preparedness within the affected population over time.

The sociocultural norms, religious beliefs, family structures and disease-related stigma present further great context-dependent challenges that may shape the implementation of precision oncology in some LMIC settings. These factors can influence the uptake of services through variable understanding, acceptance or denial of genomic information, as well as broader attitudes shaped by community perception. Where the origin of a genetic condition is understood through frameworks of fate, destiny, curse or divine judgement, this may be associated with reduced health-seeking behaviour in some communities, potentially compounding the unaddressed disease burden. Therefore, in the context of LMICs, there is a need for culturally sensitive ethical frameworks that are locally relevant rather than adopting models developed in HICs. Addressing these challenges calls for real, ongoing community involvement in how genomic testing programmes are designed and explained. The governance systems should be shaped by local voices as well as benefit-sharing arrangements that can give participating communities genuine value from the research. We are still in the infancy of adopting these safeguards.

The genetic data produced, if inadequately governed, may be repurposed for non-clinical use by law enforcement, employers or insurers for predictive risk profiling. This can cause a biased linkage of genetic information to criminal behaviour, which may contribute to unfair stereotypes about marginalised groups [8]. Therefore, structured data platforms, legal policies like the Genetic Information Nondiscrimination Act and the Health Insurance Portability and Accountability Act (HIPAA), as well as data protection measures, are required to prevent this unauthorised exploitation by external institutions. Essentially, LMICs lack such frameworks, giving birth to the stated issues. The developed world will use this data without public consent for research, while giving limited benefit to the local communities of LMICs. The World Health Organisation (WHO) has developed a set of principles with guidance from the Technical Advisory Group on Genomics (TAG-G) for the ethical collection, access, use and sharing of human genomic data [9]. These principles will guide the ethical, legal and equitable use of data, mandate transparent consent processes, foster public trust and protect human rights. LMICs may need to build local system resilience and capacity to provide equitable care, which can be made possible by fostering regional reference labs and mobile counselling services to decentralise access. The training for clinicians and ethicists focused on genetics and professional communication needs funding. Adopting international data-sharing norms such as FAIR (Findable, Accessible, Interoperable, Reusable) principles could provide a common framework for data management, thus promoting equitable international collaboration for further scientific growth [10].

Undoubtedly, PCM is essential for advancing personalised cancer therapeutics by analysing tumour genomic profiles to identify molecular targets and facilitate the development of targeted treatments. Identifying a targetable mutation does not guarantee a treatable patient. Clinical utility is shaped by tumour type, stage, assay quality, evidence strength, patient fitness, drug availability, turnaround time and resistance mechanisms, implying factors that separate biological actionability from practical treatability. A balance needs to be created regarding the diagnostic and treatment protocol in pathology, staging, surgery, radiotherapy or essential systemic treatment. Prioritisation should consider the cancer prevalence of the region, biomarker frequency, treatment benefit, affordability and whether results can alter management, while keeping in view the financial toxicity. Despite the acknowledged merits of precision oncology and its increasing global implementation, its full potential for advancing care and prevention remains unrealised. From building infrastructure to the associated costs, including research and development, testing, processing and trained personnel, a lack of alignment persists. Therefore, genomic testing should complement, not replace, basic cancer care. When resources and expertise are limited, testing should focus on cancers and biomarkers where the results are affordable, useful, and likely to change treatment rather than knowing the genetic makeup. Expertise should be established to recognise and appropriately manage potential limitations and pitfalls, including inadequate tissue samples, sequencing artefacts, variants of uncertain significance, false-positive liquid-biopsy findings due to clonal haematopoiesis, unexpected germline variants and misconceptions regarding therapeutic implications. Clear guidance should also be provided on when confirmatory germline testing and genetic counselling are warranted. Implementation success should be evaluated using measurable indicators, including the rate of actionable results, turnaround time, treatment-matching rates, clinical outcomes, financial toxicity and equity across geographic and socioeconomic groups. Administrative alignment with regulatory and payor acceptance is crucial. Health care policy can adapt to the ongoing shift to a personalised approach. It is the need of the hour to call for policies that ensure safe, equitable and context-appropriate integration of genomic technology to build public confidence and ensure ethical readiness for its appropriate implementation. An author-proposed identification of barriers, interventions and measurable outcomes is listed in Table 1.

**Table 1:** Author-proposed barrier identification, intervention and measurable outcomes LMIC: Low- and middle-income countries; FAIR: Findable, Accessible, Interoperable, Reusable.

Barriers	Example of Barrier	Intervention	Measurable Outcomes
Structural/health system	Lack of standardised clinical guidelines specific to LMICs	Targeted therapies	Rate of actionable genomic results
Socioeconomic	Low health literacy	Health care policies	Turnaround time/regular audits
Ethical	Challenges in obtaining informed consent for genomic testing	Culturally sensitive ethical frameworks	Treatment-matching rate (Time to treatment initiation)
Cultural/social	Misconceptions regarding genetic testing	Alignment with WHO principles and relevant international genomic-ethics frameworks	Clinical outcomes (Test success/failure rate) (patient-centred outcomes)
Financial	High costs of targeted therapies and companion diagnostics	Administrative alignment and testing high-risk group	Financial toxicity
Religious	Perceptions of genetic disease in relation to fate, destiny, curse, or divine judgement	Multidisciplinary and collaborative decision-making	Equity across geographic and socioeconomic groups
Legislative/regulatory	Inadequate frameworks for genomic data protection and governance	Data and digital-health	Data and governance outcomes
Disease-related	Disease-related stigma Treatment adherence challenges	Adoption of FAIR data principles	Patient-centred outcomes
Research/evidence	Limited population-specific genomic reference data	Balance between diagnostic capacity and treatment availability	Representation of LMIC populations in genomic datasets and precision-oncology research
Sustainability & Equity	Dependence on international funding and external technical expertise	Quality data handling	Equitable access to genomic testing across geographic regions
